# Modeling the *Drosophila* Gene Cluster Regulation Network for Muscle Development

**DOI:** 10.1371/journal.pone.0090285

**Published:** 2014-03-03

**Authors:** Alexandre Haye, Jaroslav Albert, Marianne Rooman

**Affiliations:** BioModeling, BioInformatics & BioProcesses Department, Université Libre de Bruxelles, Bruxelles, Belgium; University of Manchester, United Kingdom

## Abstract

The development of accurate and reliable dynamical modeling procedures that describe the time evolution of gene expression levels is a prerequisite to understanding and controlling the transcription process. We focused on data from DNA microarray time series for 20 *Drosophila* genes involved in muscle development during the embryonic stage. Genes with similar expression profiles were clustered on the basis of a translation-invariant and scale-invariant distance measure. The time evolution of these clusters was modeled using coupled differential equations. Three model structures involving a transcription term and a degradation term were tested. The parameters were identified in successive steps: network construction, parameter optimization, and parameter reduction. The solutions were evaluated on the basis of the data reproduction and the number of parameters, as well as on two biology-based requirements: the robustness with respect to parameter variations and the values of the expression levels not being unrealistically large upon extrapolation in time. Various solutions were obtained that satisfied all our evaluation criteria. The regulatory networks inferred from these solutions were compared with experimental data. The best solution has half of the experimental connections, which compares favorably with previous approaches. Biasing the network toward the experimental connections led to the identification of a model that is only slightly less good on the basis of the evaluation criteria. The non-uniqueness of the solutions and the variable agreement with experimental connections were discussed in the context of the different hypotheses underlying this type of approach.

## Introduction

Dynamical modeling of transcriptional regulation networks is an important goal of systems biology. It holds promise to understand the functioning of these networks as well as their malfunctioning, which can aid rational modification of some targeted properties. This goal is expected to be within reach due to the impressive amount of data generated during the last few years by powerful high-throughput technologies, such as DNA microarrays that provide the simultaneous expression levels of many or even all genes in a cell sample [1], [2]. Moreover, time series of DNA microarray data yield information about the evolution of gene expression levels during, for example, the developmental stages of the host organism, the response to external perturbations, or the cell cycle. If these time-dependent data were accurate and numerous enough, they would, in principle, allow the reverse-engineering of the transcriptional regulation network (see e.g. [3]–[13]). However, the mathematical model structure to be used for that purpose is unknown. Additional issues are the non-uniqueness of the parameters of the model (see e.g. [14]), the usually high level of intrinsic noise of the microarray data, and the impurity of the samples that often contain mixtures of cell types. A possibility to handle the degeneracy of the solutions is to include biology-based constraints in the modeling procedure [13]. One constraint is the robustness of the solutions with respect to parameter variations (see e.g. [13], [15]–[17]). It manages stochasticity and ensures that the overall behavior of biological systems does not vary with changes in the environment, except when large and specific perturbations come into play that lead the system to another state. The second biological constraint consists of requiring that the solutions be stable when extrapolated in time. It is indeed reasonable to assume that although the expression levels may drastically change in time, up to a few orders of magnitude, they do not become unreasonably large.

Another issue is the extremely large size of the transcription regulation network, where basically all genes of an organism are, directly or indirectly, connected. Even when large DNA microarray time series are available, the data are insufficient to identify all parameters of the model structures. Moreover, oftentimes different genes exhibit similar expression profiles, either because they are coregulated or because the noise level does not allow distinguishing them. To solve both of these problems, genes are often clustered into groups, and the modeling procedure is applied on these rather than the individual genes [9], [11], [13], [18]. The disadvantage of this approach is the lack of straightforward physical interpretation of the resulting gene cluster networks. Another approach is to consider the full transcription network of an organism as separate subnetworks that are loosely connected and can be modeled separately to a good approximation [19]–[22].

We consider in this paper *Drosophila melanogaster* as our model organism, and focus on the subset of 20 genes that are involved in muscle development during the embryonic stage. This subset has the advantage of being well described and of having available experimental data about the transcriptional interactions. To tackle modeling, we use a combination of the approaches described in the previous paragraph: we disregard the connections with genes outside this network, and cluster the genes that have similar expression profiles into classes. We then proceed to model the dynamical behavior of gene cluster expression, using coupled differential equation. To reduce the number of solutions and select those that have a biological meaning, we impose the robustness and stability constraints described above. The resulting transcriptional networks are compared to the experimental information about the transcription factor-gene interactions.

## Methods

### Experimental data on *Drosophila* genes involved in muscle development

A total of 20 genes were identified as being involved in *Drosophila* muscle development [3]. These are: CG10293 (how), CG1429 (mef2), CG17927 (mhc), CG18251 (msp-300), CG1915 (sls), CG2096 (flw), CG2328 (eve), CG2956 (twi), CG3992 (srp), CG4376 (actn), CG4677 (lmd/gfl), CG4889 (wg), CG5596 (mlcl), CG5939 (prm), CG7107 (up), CG7438 (myo31DF), CG7445 (fln), CG7895 (tin), CG9155 (myo61F), CG9885 (dpp).

The time-dependent expression profiles of these 20 genes during the embryonic development, relative to their expression in a reference sample containing a standard mixture of cells at all developmental stages, have been experimentally characterized by DNA microarray techniques [23]; they have been deposited in NCBI's Gene Expression Omnibus [24] and are accessible through GEO Series accession number GSE4347 (http://www.ncbi.nlm.nih.gov/geo/query/acc.cgi?acc=GSE4347). The DNA microarray technique [1] proceeds by extracting the mRNA from the cell sample of interest and from the reference sample, reverse transcribing them into cDNA, labeling them by two types of fluorophores, and letting them hybridize to their complementary sequences attached to a microarray. The fluorescence intensities 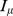 emitted by the fluorophores from the sample of interest are measured relative to the intensities 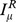 emitted by the fluorophores from the reference sample; the index 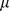 labels the mRNA molecules (or equivalently, the corresponding genes or proteins). Here we have 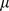 = 1,...,20. These intensities must be normalized to correct for different effects including the unequal quantities of RNA copies, differences in labeling or detection efficiencies between the fluorescent dyes, and systematic biases in the measured expression levels [25], [26]. The gene expression levels 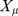 are given as the ratio of the normalized intensities 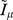 and 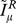, under the commonly made assumption that the RNA concentrations and fluorescence intensities are proportional [27]. Time series correspond to gene expression levels of the sample taken at 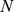 different time points 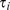 (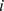 = 1,...,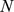). Here the time series contain 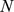 = 31 time-points and cover the 24 hours of the embryonic development, with varying sampling frequency (every 30 minutes up to time point 14 and then every hour). The time-dependent gene expression profile 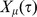 is thus defined as:
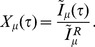
(1)


The droID database [28] lists the interactions between genes and gene products. For the 20 genes involved in *Drosophila* muscle development, 36 experimentally proven interactions are listed. These include 34 interactions between transcription factors and genes, and 3 genetic interactions, defined as interactions whose molecular mechanism is unknown or results from a cascade of interactions [29]. These interactions are listed in Table S1 of File S1. This table also contains four new interactions that were unknown when this work was started. They are used for validation purposes. Note that we overlooked protein-protein interactions because most of them are not directly obtained from experiment; rather, they are predicted from results on other species, and are thus less reliable.

### Clustering of gene expression profiles

The expression profiles 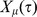 that have a similar shape are undistinguishable for modeling purposes, and we therefore cluster them into groups. However, these profiles present a high noise level and missing values. To alleviate this drawback, we first preprocess the data. Two methods were tested. The first consists of data filtering with a mobile mean procedure: 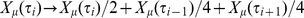; when some values are missing in this equation, they are replaced by the neighboring values. The second procedure consists of smoothing, using the cubic splines algorithm csaps of Matlab (The MathWorks Inc., Natick, MA), with parameter value 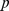 = 0.999 so that the interpolated curve follows very closely the experimental points [9]. To cluster these preprocessed expression profiles, we need to define a similarity measure and a clustering procedure. Several distances between expression profiles can be defined [30]. Since the expression levels 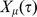 to be modeled are relative levels with respect to a gene-dependent and time-independent factor eq. (1), no difference should be made between 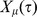 and 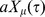, where 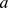 is an arbitrary positive real number. Moreover, we chose not to take into account the difference between two profiles with the same shape but different average expression levels, as such profiles are merely translated with respect to each other. We thus require a symmetric, translation-invariant and scaling-invariant distance measure with zero scaling dimension: 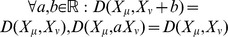, and 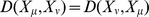. The distance satisfying these constraints has the form [30]:
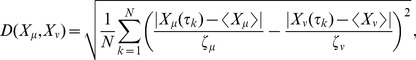
(2)in terms of the mean 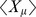 and standard deviation 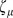:
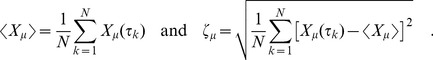
(3)


On the basis of this distance measure, the 20 genes were classified into groups displaying similar expression profiles, using two distinct clustering procedures, the k-means algorithm and a tree-like hierarchical clustering algorithm [31]. The latter proceeds by considering all profiles in separate classes and grouping them two by two, in such a way that the average distance between any pair of profiles in each class is minimum. The procedure is stopped when a threshold distance or a maximum number of classes is reached. Each of the 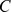 clusters so obtained, labeled by 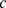 (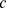 = 1,...,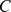), is represented by its normalized average profile, 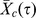. To compute this profile, we first identified the representative profile of the cluster, defined as the profile for which the distance with respect to all other members of the class is minimum. All the profiles of the cluster were then superimposed on the representative, using the translation and scaling factors that minimize the distance. The average profile corresponds to the average, at each time point, of all translated and scaled profiles in the cluster. This average profile is then normalized, by scaling and translation, so as to ensure that the new standard deviation of each profile is 1 and that the minimum expression level of all clusters is 0.

### Model structures

We assumed the system to be autonomous, and considered coupled differential equations with a transcription term and a degradation term:
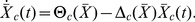
(4)where 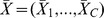 and 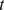 is the real, continuous time. The dot means the derivative with respect to 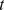. Since the transcription term 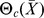 is defined to be positive, it increases the concentration 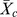 of cluster 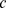, basically through the binding of transcription factors, which either activates or represses genes in this cluster. The positively defined function 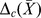, called degradation factor, describes the degradation, destabilization or activity inhibition of the gene products belonging to cluster 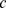, or their removal from the system. We used the model structure proposed in [13] for the full *Drosophila* gene expression time series, as it showed to be flexible and to lead to good results:
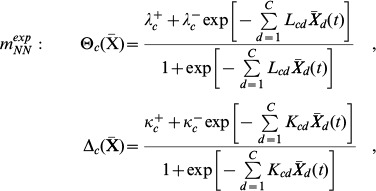
(5)with 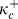, 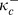, 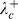, 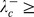
0. For defining this structure, it was assumed that the transcription term and degradation factor are modulated by interactions between genes and/or gene products. For the transcription term, these interactions represent the binding of activating or repressing transcription factors as well as the whole cascade of protein-protein interactions occurring before the binding of the transcription factors. For the degradation term, these interactions tend to either prolong ( *e.g.* through stabilizing complexes) or shorten ( *e.g.* through degradation by proteases) their period of activity. The parameters 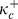 and 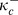 (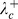 and 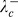) symbolize the maximum and minimum degradation rate (transcription rate) when 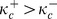 (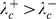) and the converse when 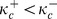 (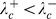), and 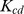 and 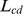 give the influence (stabilizing or destabilizing according to their sign) of gene (product) 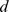 on gene (product) 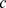.

Two other model structures were also tested, which are particular cases of the first. These are:
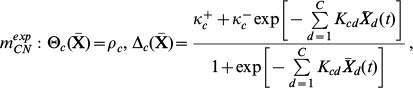
(6)which is obtained from (5) by posing 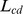 = 0, 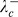 = 0 and 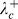 = 2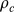, and
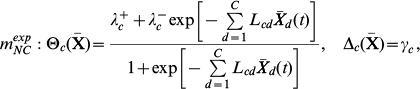
(7)obtained from (5) by posing 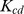 = 0, 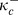 = 0 and 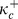 = 2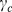.

### Parameter identification

The gene expression network was built iteratively by increasing the connectivity 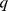 which is defined as the average number of connections ending at a node (or class). In a first stage, the number of connections was considered to be identical for all nodes. The procedure starts by considering 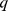 = 1, and determines, for each node, the connection (defined by a series of parameters) that minimizes an objective function. It continues by incrementing 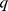 until it is large enough to get sufficiently small values of the objective function.

A two-step procedure, based on two different objective functions, was used for parameter identification so as to manage the large amount of parameters and the non-linearity of the equations. The first step consists of constructing the network by reproducing the derivatives of the gene expression levels rather than the gene expression levels themselves. The objective function 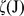, where 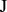 denotes generically all parameters of the model, is thus the square root of the square difference of the measured and estimated expression level derivatives, summed over all time points:
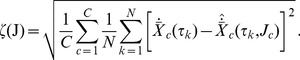
(8)


This entails considering the expression levels and their derivatives as independent variables and reducing the identification to an algebraic problem. Details of this procedure can be found in [13]. In the second step, the connections defined in the first stage for 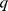 = 1, 2,... are maintained and the parameters of these connections are identified so as to minimize another objective function, expressed as a function of the difference between measured and estimated profiles rather than their derivatives. Two variants of this objective function are considered, 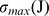 and 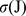. They are defined as:
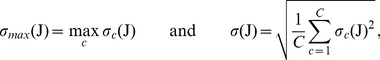
(9)


where
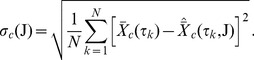
(10)


The estimate of the gene expression profiles, 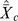, is obtained by integration of the differential equations (4), using one of the model structures given by eqs (5–7), and the ode45 routine of Matlab. The parameters 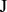 are identified so as to minimize either 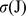 or 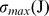, using Matlab's fmincon optimization algorithm. The initial values of the parameters are set to those obtained for the 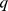-1 identification, with the newly added parameters set to zero.

### Parameter reduction

The next step consists of eliminating unnecessary parameters among 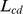 and 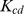 which appear in eqs (5–7), while requiring that at least one connection per gene class be kept. We proceed by dropping one parameter at a time at each step in the iteration, according to two criteria:

1. the parameter of smallest absolute value; this procedure is referred to as 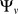;
2. the parameter which, when dropped, leads to the smallest increase of 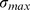; this procedure is called 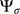.

These criteria turned out to be more effective than those based on the Fisher information matrix [13]. After a parameter is eliminated the remaining parameters are optimized again using the local optimization algorithm fmincon from Matlab. The elimination procedure is then reiterated.

### Evaluation of the solutions

Four criteria were used to evaluate the quality of the estimated profiles:

1. the number or remaining parameters;
2. the standard deviations 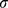 and 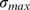 between estimated and experimental profiles, defined in eq. (9);
3. the robustness of the solution with respect to perturbations of its parameters; this is estimated by adding to each parameter in turn 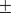
1% of its value, determining which perturbation leads to the largest deviation between measured and estimated expression levels, 
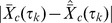
, for any cluster c and time point 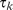, and computing the value of the standard deviation 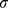 obtained with this perturbed parameter, denoted 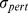;
4. the stability of the solution, evaluated by extrapolating the estimated profiles up to a time 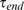 and by computing the difference between the average value of the estimated gene expression levels over the measuring period and the extrapolated level:

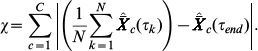
(11)


The time 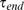 is taken to be 3 times the measured (embryonic) time span.

## Results

### Clustering of the gene expression profiles

The raw data points representing the gene expression levels 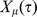 of the 20 genes involved in the embryonic muscle development of *Drosophila*, were first preprocessed to fill in the missing points and to decrease the effect of measurement noise, as described in Materials and Methods. Two procedures were tested, consisting of filtering and/or smoothing. Moreover, given that some of the expression profiles have very similar shapes and are thus basically indistinguishable, we proceed to cluster them into groups. Since the profiles are defined up to a gene-dependent factor (see eq. (1)), the distance used to evaluate the similarity is a translation and scaling-invariant measure of scaling dimension zero, denoted 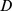 and defined in eq. (2). Two different classification methods were tested with this distance, *i.e.* k-means and a tree-like clustering algorithm (see Methods section).

To choose the most appropriate clustering and preprocessing method, we computed: 1) the average distance 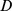 (defined by eq. (2)) between the members of the same class; 2) the average distance between members of different classes; 3) the average distance 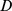 between the representative member of a class (defined in Methods) and the other members; and finally 4) the average distance between the representative members of different classes. To have well-defined classes, the first and third distance measures that correspond to intraclass distances must be as low as possible, while the second and fourth distance measures must be as high as possible.

The results are given in Table 1 for classifications into 10 classes and in Table S2 in File S1 for 5–15 classes. Preprocessing the data by successively filtering and smoothing decreases all the distances in general, and decreases even more the intra- than the interclass distances. We thus selected this preprocessing procedure. The lowest intraclass distances and the highest interclass distances are sometimes obtained with the tree-like clustering procedure and sometimes with k-means, depending on the number of classes. However, k-means performs more often slightly better and we thus selected it as clustering procedure. The choice of the number of classes is somewhat arbitrary as we do not see a gap in the intra- and interclass distances when decreasing the number of classes (Table S2 in File S1).

**Table 1:** Effect of the preprocessing procedure and clustering algorithm on the quality of the clusters.

Preprocessing	Classification	Intraclass	Intraclass	Interclass	Interclass
procedure	method	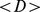	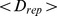	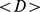	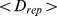
Filtering	tree-like	0.43	0.39	1.05	1.06
	k-means	0.41	0.31	1.01	1.08
Smoothing	tree-like	0.33	0.29	1.00	1.00
	k-means	0.29	0.23	0.94	1.00
**Filtering &**	tree-like	0.31	0.29	0.94	0.99
**Smoothing**	**k-means**	**0.28**	**0.23**	**0.95**	**1.03**

The number of classes is set to 10. The optimal procedure is indicated in bold. Intraclass 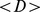: average distance between members of the classes; Intraclass 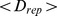: average distance between members of the classes and their representative member; Interclass 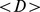: average distance between members of different classes; Interclass 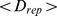: average distance between representative members of different classes.

To choose the total number of classes, we were guided by: the concern for having basically indistinguishable profiles in the same class; different profiles in different classes; and a sufficiently small number of classes to ensure that the parameters can be reliably identified from the available data. The analysis of Table S2i File S1 indicates that the average distance between members of the classes falls below 0.3 when clustering into 10 classes and more. A visual inspection of the superimposed expression levels in the classes confirmed that these 10 classes are well-defined (see Fig. S1 in File S1). We thus fixed the number of classes to 10. This classification grouped together the genes {*mhc*, *mlc1*, *prm*, *up*, *myo31DF*, *myo61F*}, {*msp-300*, *sls*}, {*actn*, *wg*, *tin*, *dpp*} and {*eve*, *twi*}; all other classes contain a single gene.

**Figure 1:**
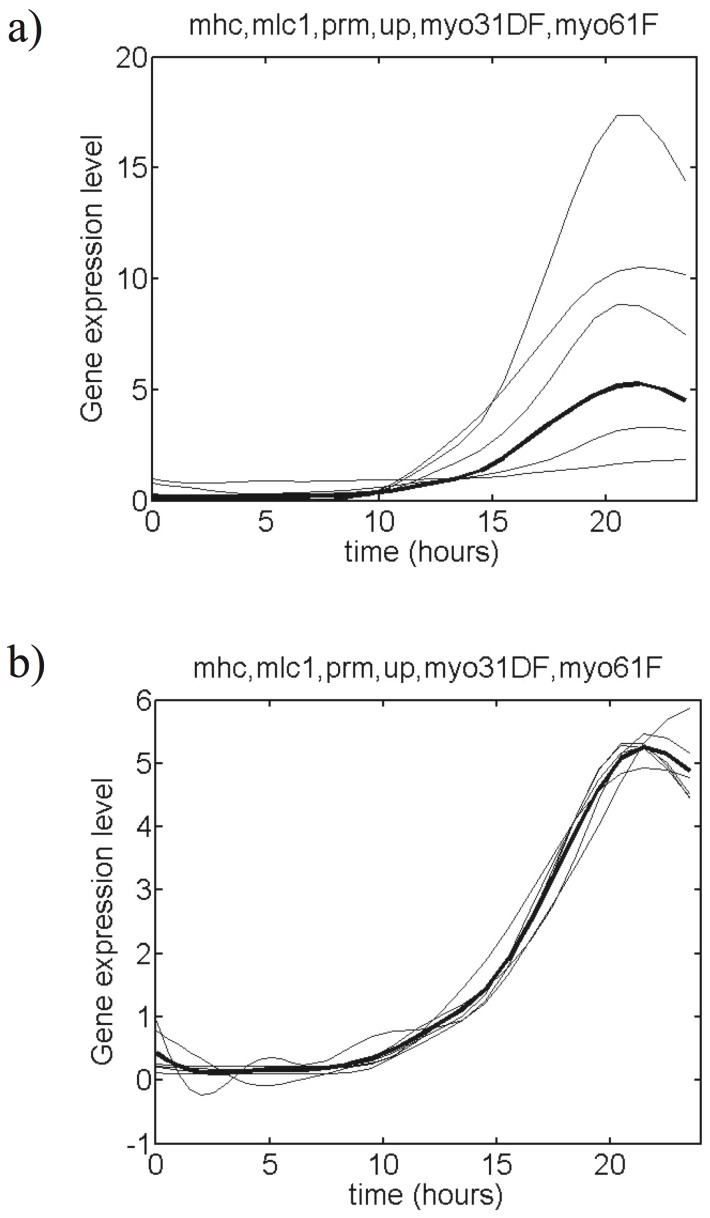
*Drosophila* muscle gene expression profiles belonging to a cluster. The 6 members of the cluster are listed at the top of the figure. The results for all clusters are given in Figs S1-S2 of File S1. (a) Filtered and normalized gene expression profiles contained in the cluster; the representative profile is depicted in bold. (b) Expression profiles superimposed onto the representative profile by translation and scaling; the average profile is depicted in bold.

The representative and other members of these 10 classes are shown in Fig. 1 and in Fig. S1 of File S1. Each of these clusters labeled by 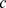 (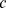 = 1,...,10) is represented by its normalized average profile, 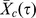, which is defined in the Methods section and is depicted in Figs. 1, and S1 and S2 in File S1.

### Dynamical modeling of gene expression profiles

The time-evolution of the 10 normalized average expression levels, 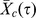, of the 20 genes involved in muscle development was modeled using an autonomous model structure (eq. (4)) with three versions of the transcription term and degradation factor given by eqs (5–7).

Because of the large number of parameters and the nonlinearity of the equations, it is impossible to have a reliable direct identification of all the parameters that define all the possible connections between genes. So, assuming that the real gene expression network is sparse, we first determine the necessary connections, assuming a constant connectivity 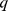 for all nodes (see section 2.4 for details). We start from 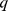 = 1 and increase it until the value of the objective function is sufficiently small. Moreover, we use successively two objective functions, denoted 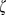 and 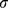, defined in eqs (8–9). The first is given as a function of the difference between the derivatives of the experimental and estimated profiles, and the second as a function of the difference between the experimental and estimated profiles. The first is used to define the important connections and the second to optimize the parameters.

Moreover, two variants of the latter objective function are used: 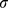 which is the standard deviation between estimated and experimental profiles averaged over all classes, and 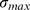 which is the largest standard deviation of all classes (eq. (9)). Using the former has the drawback that some classes may be modeled very well and others very poorly. Using the latter ensures that all classes have 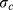 values lower than 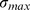, and gives thus slightly more homogenous and satisfactory results. Hence we keep 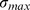 as the objective function.

**Figure 2:**
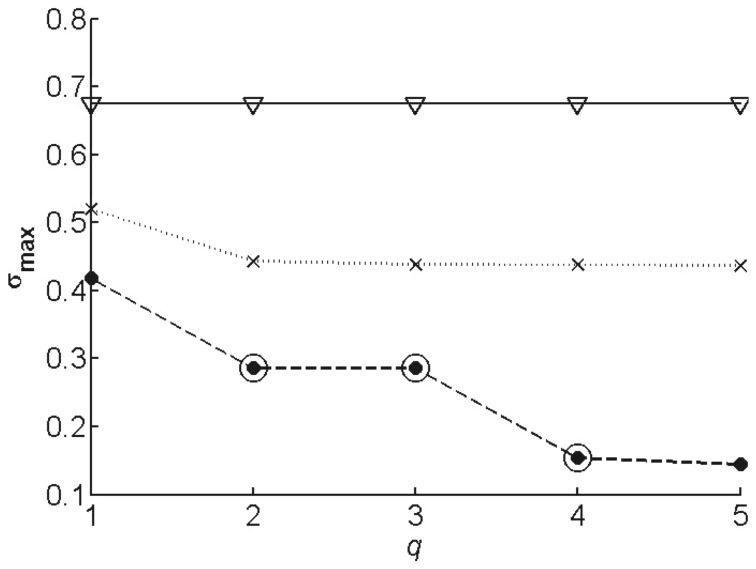
Value of the objective function 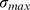 as a function of the connectivity 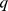, for different model structures. The results obtained with the model structure 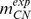 are represented by a solid line with triangles, with 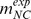 by a dotted line with crosses, and with 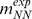 by a dashed line with dots. The circled points indicate the solutions selected for parameter reduction.

**Figure 3:**
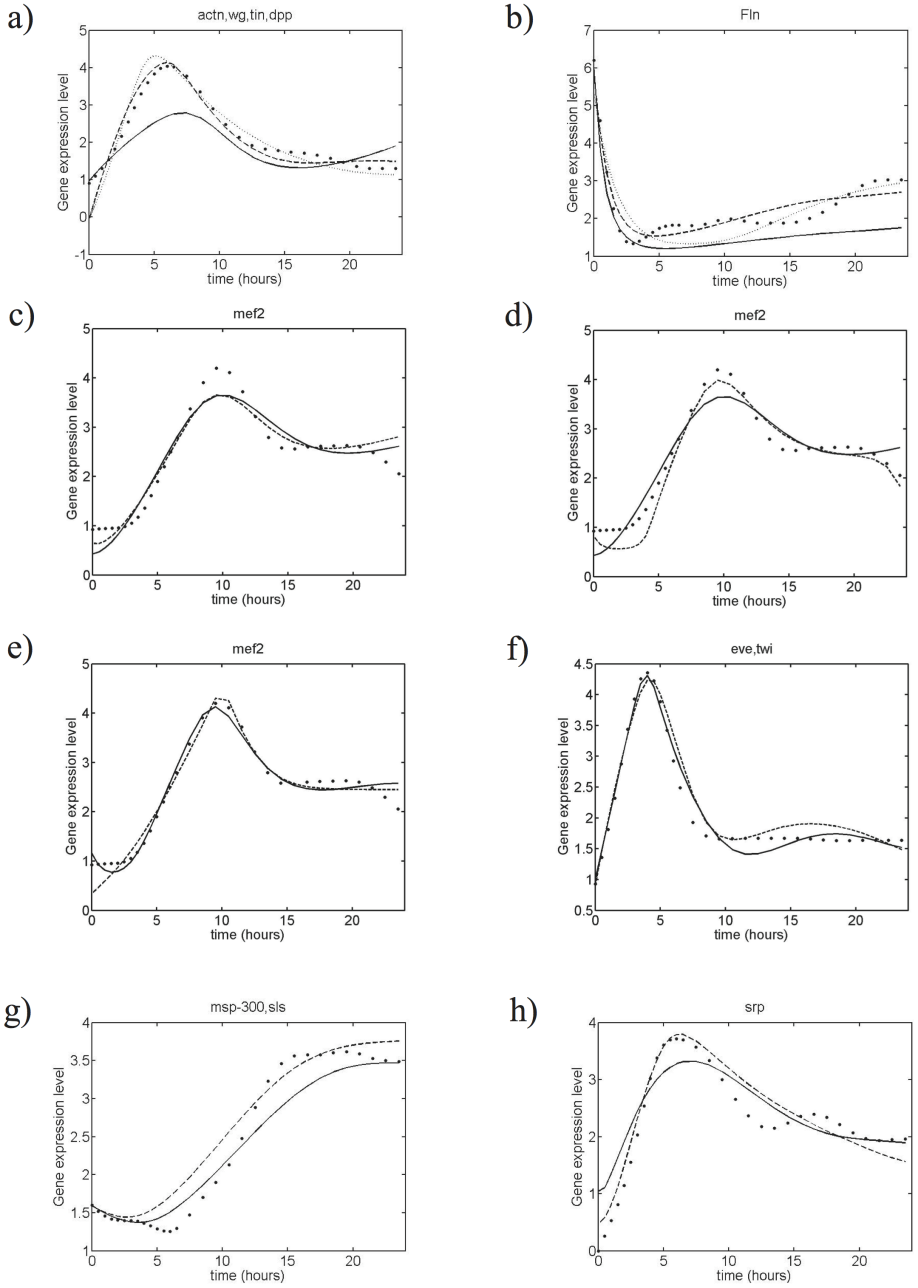
Estimated and experimental expression profiles for a few clusters. The results for all clusters are given in Figs S3-S7 of File S1. Dots: clustered, filtered and smoothed experimental data; (a)-(b): Estimated expression profiles for clusters {*actn*, *wg*, *tin*, *dpp*} and {*fln*} using the three model structures; dashed line: 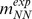 ; dotted line: 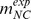 ; solid line: 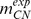; (c)-(f): Estimated expression profiles for cluster {*mef2*} and {*eve*, *twi*} using the model structure 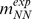 and 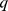 = 2 (c), 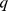 = 3 (d) and 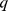 = 4 (e-f); solid line: before parameter reduction; dashed line: after parameter reduction using the 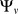 procedure; (g)-(h): Estimated expression profiles for clusters {*msp-300*, *sls*} and {*srp*} using the model structure 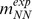, 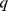 = 3, and the biasing procedure towards the experimental network; solid line: before parameter reduction; dashed line: after parameter reduction using the 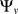 procedure.

The evolution of 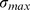 during the network construction, from connectivity 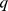 = 1 to 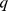 = 5, is shown in Fig. 2. Clearly, the model structure that has the largest number of parameters, 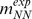, in which neither the transcription term nor the degradation factor is constant, is superior to the other two structures. For this structure, the minimal connectivity required (for which the 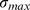 value is reasonably low) is equal to 2, but a connectivity of 4 yields much lower values of 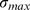. Figs. 3a-b and Fig. S3 in File S1 illustrate the superiority of 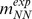: only this structure allows the correct reproduction of the data. We restrict ourselves to this model structure and connectivity in the following.

To get rid of the unnecessary connections, we proceed to parameter elimination. Two methods were tested: 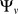 and 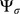, where the eliminated parameters are those of smallest absolute value or those that lead to the smallest increase of the objective function 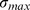 (see Methods). Note that when several parameter eliminations lead to the same value of 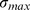, the one that increases 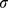 the least was chosen. The elimination procedure was performed until a threshold valued of 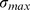 was reached. This threshold was set at 0.3, by visual inspection, so as to ensure a fair reproduction of the experimental profiles. Moreover, in order to mimic biological reality, we selected solutions that were robust against perturbations of the parameters; in particular we required the standard deviation 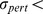
0.5 (see Methods). We did not put a threshold on the stability of the solution, estimated by 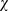 (eq. (10)).

Note that these two characteristics, robustness and stability, are quite important for modeling biological systems. Indeed, all such systems have a stochastic behavior that depends, among others, on changes in the environment, the amount of biomolecules, their possible binding and function. However, these changes do not affect the main properties of the system, which continues to give similar responses to similar stimuli. Only very large or very specific perturbations can bring the system out of its correctly functioning state and lead it to another state, which can be functional or dysfunctional, depending on the perturbation. It is thus very important that the models that simulate biological systems have the same properties, and thus do not yield very different solutions for similar parameter values. The other characteristic of biological systems is its stability. Even though the available data usually cover only a part of the system's life, it is reasonable to assume that the expression levels continue to be of the same order of magnitude, never becoming unrealistically large or negative. The same property is expected to be built in the model: the solutions must take realistic values until the next perturbation or developmental stage, or the end of the organism's life.

The results of the elimination procedures 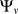 and 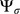 for 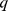 = 2–4 are given in Table 2 and Table S3 in File S1. These two procedures gave comparable results for the data reproduction, and 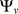 gives usually better results for the stability and robustness, especially for 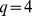. We will thus in the following only detail the results obtained with 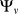.

**Table 2:** Characteristics of the full and reduced solutions using the model structure 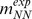.

Model	q	Solution	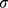	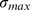	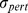	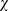	NC 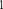	PC 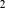	AC 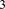
	2	full	0.29	0.29	0.43	3.02	20	5/17(29%)	68/83 (82%)
		reduced	0.27	0.27	0.30	13.78	20	5/17(29%)	68/83 (82%)
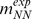	3	full	0.28	0.29	0.43	3.01	30	7/17(41%)	60/83 (72%)
		reduced	0.28	0.29	0.43	0.95	20	4/17(24%)	67/83 (81%)
		full	0.15	0.15	0.43	3.01	40	8/17(47%)	51/83 (61%)
		**reduced**	**0.20**	**0.21**	**0.39**	**2.77**	**29**	**8/17(47%)**	**62/83 (75%)**
Biased 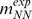	3	full	0.45	0.46	0.73	1.68	30	17/17(100%)	70/83 (84%)
		**reduced**	**0.31**	**0.33**	**1.12**	**3.21**	**27**	**17/17(100%)**	**73/83 (88%)**

**Figure 4:**
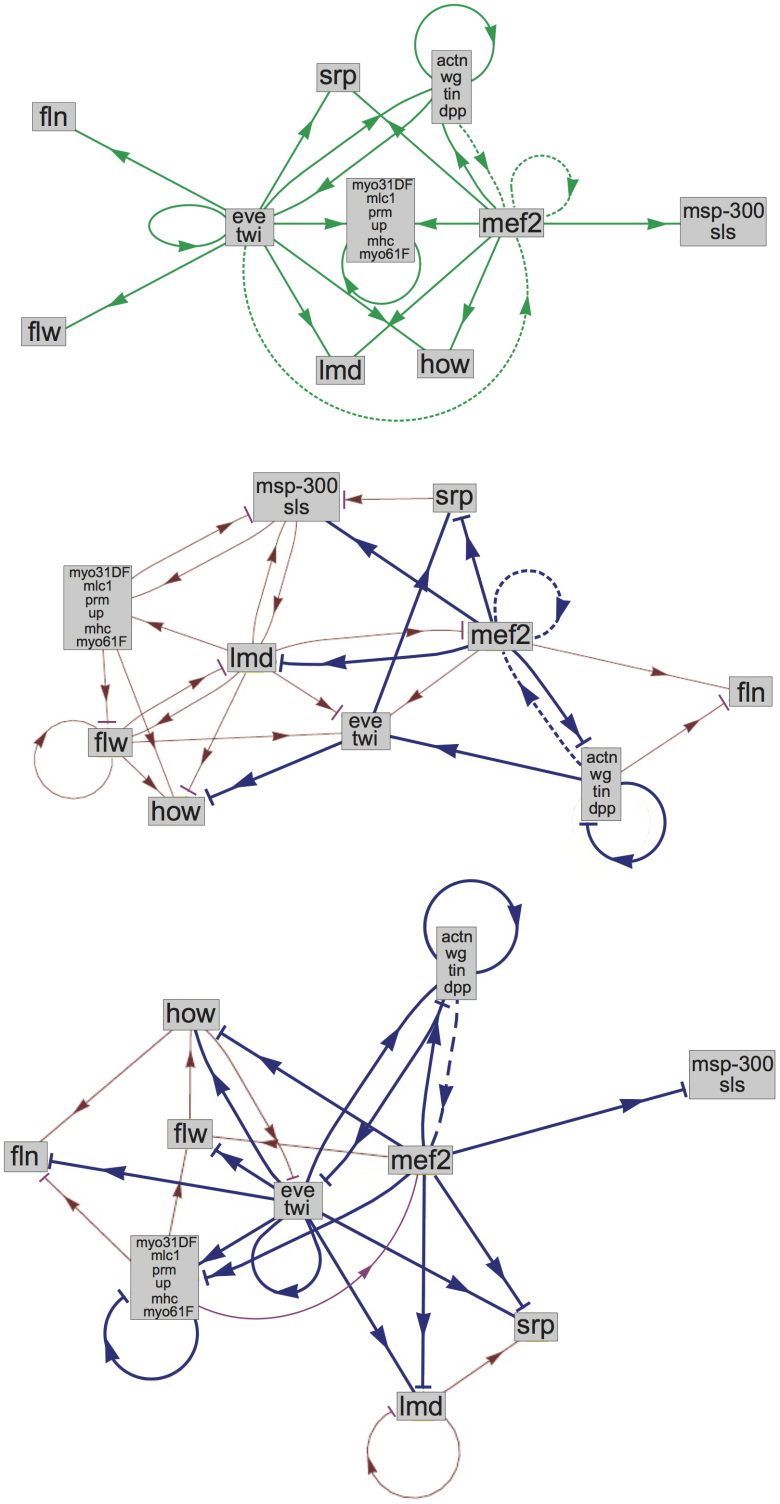
Gene regulation networks. (a): Experimental connections; the dashed connections correspond to the 3 connections that were unknown when this work was performed; (b): Network obtained with the model structure 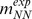 and 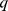 = 4, after parameter reduction using the 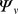 procedure; (c): Network obtained with the model structure 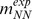, 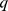 = 3 and the biasing procedure towards the experimental network, after parameter reduction; (b-c): the connections in blue are the experimental connections that have been predicted; the blue dashed connections correspond to the new experimental connections that have been predicted.

The last two lines contain the solutions biased towards the experimental network. The networks corresponding to the solutions in bold are depicted in Fig. 4b-c. 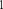
NC : number of connections in the estimated network; 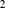
PC: fraction of these connections that are among the 17 experimentally verified connections (see Table S1 in File S1); 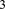
AC: fraction of the non-connections that are not among the 17 experimentally verified connections (thus that are among the 10×10–17 = 83 experimental non-connections).

The estimated profiles are shown in Figs 3c-f and Figs S4-S6 in File S1. The number of connections of the reduced solutions vary between 20 (for 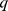 = 2–3) and 29 (for (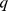 = 4) and, accordingly, the reproduction of the experimental profiles is somewhat better for 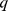 = 4 (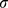 and 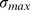 = 0.2) than for 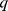 = 2–3 (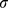 and 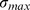 = 0.3). Note that 20 seems to be the minimum number of connections needed: the reduction procedure applied on the 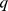 = 2 solution, with 20 initial connections, fails to eliminate further connections. All the solutions show a fairly good robustness with respect to the variations of the parameters, with values of 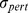 between 0.3 and 0.4. In contrast, the stability upon extrapolation in time differs among the solutions. The best reduced solution is that obtained from 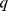 = 3 (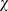 = 0.9), followed by the 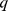 = 4 solution (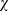 = 2.8); the 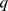 = 2 solution is not stable at all (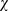 = 13.8).

### Comparison with the experimental network

The connections between gene clusters obtained by our dynamical modeling procedure can be compared to the experimentally determined connections, described in section 2.1 and Table S1 in File S1. For this comparison, we first have to transform the experimental network between individual genes into a network between gene clusters. This is done by considering two clusters as being connected if at least one member in each cluster is connected to at least one member in another cluster. The experimental cluster network so defined contains 17 connections; it is shown in Fig. 4a. Note that these interactions are oriented but unsigned, as the sign is not experimentally determined.

The intersection between the experimental and modeled networks is indicated in Table 2. The smallest intersection is obtained with the reduced 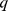 = 3 solutions (only 4 out of 17 reproduced connections), whereas the largest intersection is obtained with the full and reduced 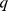 = 4 solutions (8 out of 17, which amounts to 47%). The number of experimentally non-observed connections that are also absent in the modeled solutions is of course much larger (between 61% and 82%).

The number of common connections between the experimental network and the different solutions can be compared to the number of common connections that are expected at random. The most significant result is found for the 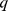 = 4 reduced solution: the probability of finding 8 common connections among two sets of 29 and 17 connections each, out of a total set of 100 connections, is equal to 0.048. The same value is found for the absent connections.

The network corresponding to this best solution (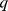 = 4 reduced solution) is depicted in Fig. 4b, with the correctly reproduced connections highlighted. Among the 8 connections that are in agreement with experiment, four involve the gene *mef2* (myocyte enhancing factor 2). In particular, the subnetwork involving the genes and gene clusters *mef2*, *srp*, *lmd*, *how*, {*eve*, *twi*} and {*actn*, *wg*, *tin*, *dpp*} is in good agreement with experiment.

Note moreover that all existing functional connections have not yet been determined experimentally. Therefore some of the predicted connections may in fact be real ones. This is indeed the case: among the four new connections that were unknown when this work was performed (indicated in Table S1 in File S1), which correspond to three new connections between clusters (see Fig. 4a), two were actually predicted, as shown in Fig. 4b. These correctly predicted connections involve *mef2*, which supports the conclusion that this region of the network is well reproduced by our model. Adding these new connections increases the number of correctly predicted connections to 10, out of a total of 20 experimental connections.

Our gene regulation network connects gene clusters rather than individual genes and is thus of a different type than the networks obtained with other methods. Nevertheless, the fraction of correctly predicted connections, either between genes or gene clusters, can be compared among the different methods applied to the same ensemble of *Drososphila* muscle development genes [19], [20]. These methods do not reach our 50% score.

### Biasing towards the experimental network

To analyze if it is possible to find solutions that reproduce the data well but are different from those obtained in the previous section and are more consistent with the experimental data, we perform a biased modeling procedure. This procedure follows the same two steps: first the construction of the network from 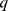 = 1 to higher 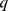 by minimizing the cost function 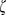 (eq. (8)), and then the minimization of the cost function 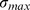 (eq. (9)) while keeping the same network. However, here, instead of allowing a free choice among all possible connections, the choice was biased towards the experimentally proven connections: if, for a given cluster, experimental connections do exist and have not yet been included in the network in a previous step, the choice is limited to those; otherwise the choice is free. Moreover, in the parameter reduction procedure, parameters involved in the experimental connections may be eliminated but the connection may not be dropped entirely.

The results obtained with this procedure are given in the last two lines of Table 2 and in Figs 3g-h and Fig S7 in File S1. The reproduction of the expression profiles is somewhat less accurate than with the unbiased method but remains good, with 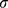 = 0.4 and 0.3 for the full and reduced solutions starting at 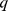 = 3. Note that the optimization of the solutions performs less well with some imposed connections, as 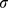 is higher for the full than for the reduced solution. The robustness of the reduced solution is also somewhat less good than for the unbiased procedure (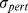 = 1.1), whereas the stability is similar. Note that the total number of connections in the reduced solution is equal to 27, and that we had to add 10 connections in addition to the 17 experimental ones to reach a reasonable accuracy in the profile reproduction. However, the number of 27 connections is comparable to the number of connections obtained with the unbiased procedure (20–29). Note that among the three new experimental connections that were not imposed in this procedure, one appears to be correctly predicted.

## Conclusion

One successful result of our work is the consistent construction of dynamical models, on the sole basis of the gene expression profiles of the genes involved in *Drosophila* muscle development obtained from DNA microarray series. The models obtained reproduce the expression profiles quite well, are robust against parameter variation and do not take unrealistically large values when extrapolated in time. However, our results present two important drawbacks. First, the solutions are not unique, and different networks are obtained with very similar data reproduction abilities. The additional requirement of robust and stable solutions filters out some of them, but the number of acceptable solutions remains high. Second, half of the experimental connections are obtained by the best of our unbiased models. To obtain all experimental connections, we had to bias the model construction towards the experimental network.

The amount of 50% of the experimental connections found by our models compares quite favorably with the results of other analyses. However, our solution is still far from perfect and it is worthwhile to question the basics of our approach. We made a number of assumptions that, although commonly made in biological modeling, could explain the limited overlap between estimated and experimental connections. These assumptions are detailed hereunder.

• We considered only the 20 genes known to be involved in muscle development of *Drosophila*. In reality, these genes are connected to other genes. We suppose here that these additional connections are not (or much less) important for the transcriptional regulation of these 20 genes. More generally, we disregarded the effect of all external factors on the regulation of these 20 genes, which is quite a bold (but common) assumption.

• As some of these 20 genes have similar experimental expression profiles, which are moreover quite noisy, we preprocessed the data by filtering and smoothing them, and grouped the similar profiles together using a k-means classification procedure and a translation-invariant and scaling-invariant distance measure. Although we tested several preprocessing and classification procedures and although the selected ones appear to perform quite well, we cannot be sure that the noise is eliminated in the right way, and that the clusters are formed adequately.

• As some genes were clustered together, the transcriptional model we derive connects gene clusters instead of individual genes. The interpretation is that when a cluster is found to regulate another cluster, some of their members do so, but not necessarily all. It is indeed possible that the different members of a cluster – even though their expression profiles are similar – are not regulated by the same gene (cluster). The different – almost equivalent – solutions in terms of data reproduction and robustness that we found could well reflect the differences in connections between individual members of the clusters.

• Another possibility is that the DNA microarray data, and thus the networks predicted from them, correspond to external conditions that differ from those of the experimental inter-gene connections. It has for example been shown that networks may be different when responding to different types of stress [32].

• The model structure we selected is quite flexible and gives good results in terms of data reproduction; it could however be argued that it does not mimic the biological mechanism and that another model structure should be used.

• The experimentally determined interactions are listed as regulatory interactions. However, some of them could be involved only indirectly in regulation, and others could be side actors. Moreover, not all interactions are known today, and some of the predicted interactions – or of the non-predicted ones – will perhaps be experimentally demonstrated in the future.

It is difficult at this point to identify the reason for the limited – though substantial – overlap between experimental and estimated connections and of the large number of almost equivalent solutions. Note that the latter result could be taken as a positive result that mimics reality. Indeed, gene regulation networks have been shown experimentally to display some elasticity [33].

The results of our analysis indicate that more extensive and more specific experimental data is needed to decide between the different hypotheses. For example the existence of connections predicted by our models, depicted in Fig. 4b-c, should be verified experimentally. In particular the additional connections involving the *mef2* gene in Fig. 4b, which is at the center of a quite well predicted subnetwork, are interesting candidates to be tested.

## Supporting Information

File S1
**Supporting tables and figures.** Table S1: The interactions between the 20 genes involved in muscle development that have been observed experimentally. Table S2: Effect of the clustering algorithm and the number of clusters on the quality of the clusters. Table S3: Characteristics of the full and reduced solutions using the model structure 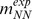 and the reduction procedure 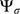. Figure S1: The four clusters of Drosophila muscle gene expression profiles containing more than one member. Figure S2: The average profile of the ten clusters. Figure S3: Experimental and estimated gene expression profiles, with q = 3. Figure S4: Experimental and estimated gene expression profiles for model 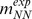 with 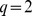 before and after parameter reduction using the 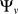 procedure. Figure S5: Experimental and estimated gene expression profiles for model 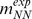 with 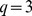 before and after parameter reduction using the 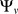 procedure. Figure S6: Experimental and estimated gene expression profiles for model 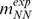 with 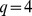 before and after parameter reduction using the 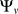 procedure. Figure S7: Experimental and estimated gene expression profiles for model with 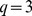 before and after parameter reduction using the 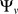 procedure, when the 17 experimentally validated connections are imposed.(PDF)
