# Supplementary material for: Modeling the Drosophila Gene Cluster Regulation Network for Muscle Development
Source: PLoS One. 2014 Mar 3;9(3):e90285. doi: 10.1371/journal.pone.0090285 (PMC3940846; doi:10.1371/journal.pone.0090285)
Supplement: File S1 — Supporting tables and figures. Table S1: The interactions between the 20 genes involved in muscle development that have been observed experimentally. Table S2: Effect of the clustering algorithm and the number of clusters on the quality of the clusters. Table S3: Characteristics of the full and reduced solutions using the model structure and the reduction procedure . Figure S1: The four clusters of Drosophila muscle gene expression profiles containing more than one member. Figure S2: The average profile of the ten clusters. Figure S3: Experimental and estimated gene expression profiles, with q = 3. Figure S4: Experimental and estimated gene expression profiles for model with before and after parameter reduction using the procedure. Figure S5: Experimental and estimated gene expression profiles for model with before and after parameter reduction using the procedure. Figure S6: Experimental and estimated gene expression profiles for model with before and after parameter reduction using the procedure. Figure S7: Experimental and estimated gene expression profiles for model with before and after parameter reduction using the procedure, when the 17 experimentally validated connections are imposed. (PDF) [file pone.0090285.s001.pdf]

Supporting Information

Modeling the *Drosophila* gene cluster regulation network for muscle development

Alexandre Haye, Jaroslav Albert, Marianne Rooman

BioModeling, BioInformatics & BioProcesses, Université Libre de Bruxelles, Belgium

Table S1. *The interactions between the 20 genes involved in muscle development that have been observed experimentally and are stored in the droID database (http://www.droidb.org/); they include transcription factor (TF)-gene interactions and genetic interactions. The genes in columns 3 and 4 regulate the genes in column 2. The genes in red correspond to new connections that were not taken into account in building the networks; they are used only for validation purposes.*

CG number	Gene symbol	TF-gene interactions	Genetic interactions
CG10293	how	mef2, twi	
CG1429	mef2	mef2, tin, twi	twi
CG17927	mhc	mef2	up
CG18251	msp-300	mef2	
CG1915	sls	mef2	
CG2096	flw	twi	
CG2328	eve	eve, twi, tin	
CG2956	twi	tin, twi	
CG3992	srp	mef2, eve, twi	
CG4376	actn	twi	
CG4677	lmd or gfl	mef2, twi	
CG4889	wg	mef2, twi, tin	twi, dpp
CG5596	mlc1	mef2	
CG5939	prm	twi	
CG7107	up	mef2, twi	
CG7438	myo31DF	mef2, twi	
CG7445	fln	twi	
CG7895	tin	mef2, eve, twi, tin	
CG9155	myo61F	mef2	
CG9885	dpp	mef2, twi, tin	

Table S2. *Effect of the clustering algorithm and the number of clusters on the quality of the clusters. The gene expression profiles were preprocessed by filtering and smoothing. Intraclass <D>: average distance between members of the classes; Intraclass <D_{rep}>: average distance between members of the classes and their representative member; Interclass <D>: average distance between members of different classes; Interclass <D_{rep}>: average distance between representative members of different classes.*

Number of classes	Classification method	Intraclass <D>	Intraclass <D_{rep}>	Interclass <D>	Interclass <D_{rep}>
5	tree-like	0.43	0.36	1.04	1.07
	k-means	0.45	0.36	1.01	1.03
6	tree-like	0.40	0.34	1.03	1.10
	k-means	0.42	0.32	1.01	1.06
7	tree-like	0.38	0.33	1.01	1.06
	k-means	0.35	0.30	1.00	1.06
8	tree-like	0.33	0.30	0.95	1.05
	k-means	0.33	0.28	0.98	1.04
9	tree-like	0.32	0.30	0.94	1.02
	k-means	0.30	0.25	0.96	1.02
10	tree-like	0.31	0.29	0.94	0.99
	k-means	0.28	0.23	0.95	1.03
11	tree-like	0.31	0.27	0.95	0.96
	k-means	0.28	0.21	0.95	1.01
12	tree-like	0.28	0.26	0.93	0.96
	k-means	0.27	0.19	0.94	0.98
13	tree-like	0.26	0.23	0.91	0.95
	k-means	0.25	0.17	0.94	0.96
14	tree-like	0.26	0.19	0.95	0.94
	k-means	0.21	0.15	0.93	0.95
15	tree-like	0.16	0.12	0.92	0.94
	k-means	0.16	0.12	0.93	0.94

Table S3. *Characteristics of the full and reduced solutions using the model structure* $m_{NN}^{\exp}$ *and the reduction procedure* Ψ_σ. *This Table differs from Table 2 in main text by the reduction procedure which is* Ψ_σ *here and* Ψ_ν *in Table 2.* [1]*NC : number of connections in the estimated network;* [2]*PC: fraction of these connections that are among the 17 experimentally verified connections (see Table S1);* [3]*AC: fraction of the non-connections that are not among the 17 experimentally verified connections (thus that are among the 10x10-17=83 experimental non-connections).*

q	Solution	σ	σ_{max}	σ_{pert}	χ	NC[1]	PC[2]	AC[3]
2	full	0.29	0.29	0.43	3.02	20	5 /17 (29%)	68/83 (82%)
	reduced	0.24	0.25	0.55	1.00	20	2/17 (12%)	65/83 (78%)
3	full	0.28	0.29	0.43	3.01	30	7/17 (41%)	60/83 (72%)
	reduced	0.22	0.23	0.80	0.95	21	2/17 (12%)	64/83 (77%)
4	full	0.15	0.15	0.43	3.01	40	8/17 (47%)	51/83 (61%)
	reduced	0.22	0.23	1.63	74.63	29	6/17 (35%)	60/83 (72%)

Figure S1. *The four clusters of Drosophila muscle gene expression profiles containing more than one member. Left hand side: filtered and normalized gene expression profiles contained in the cluster; the representative profile is depicted in bold. Right hand side: expression profiles superimposed onto the representative profile by translation and scaling; the average profile is depicted in bold.*

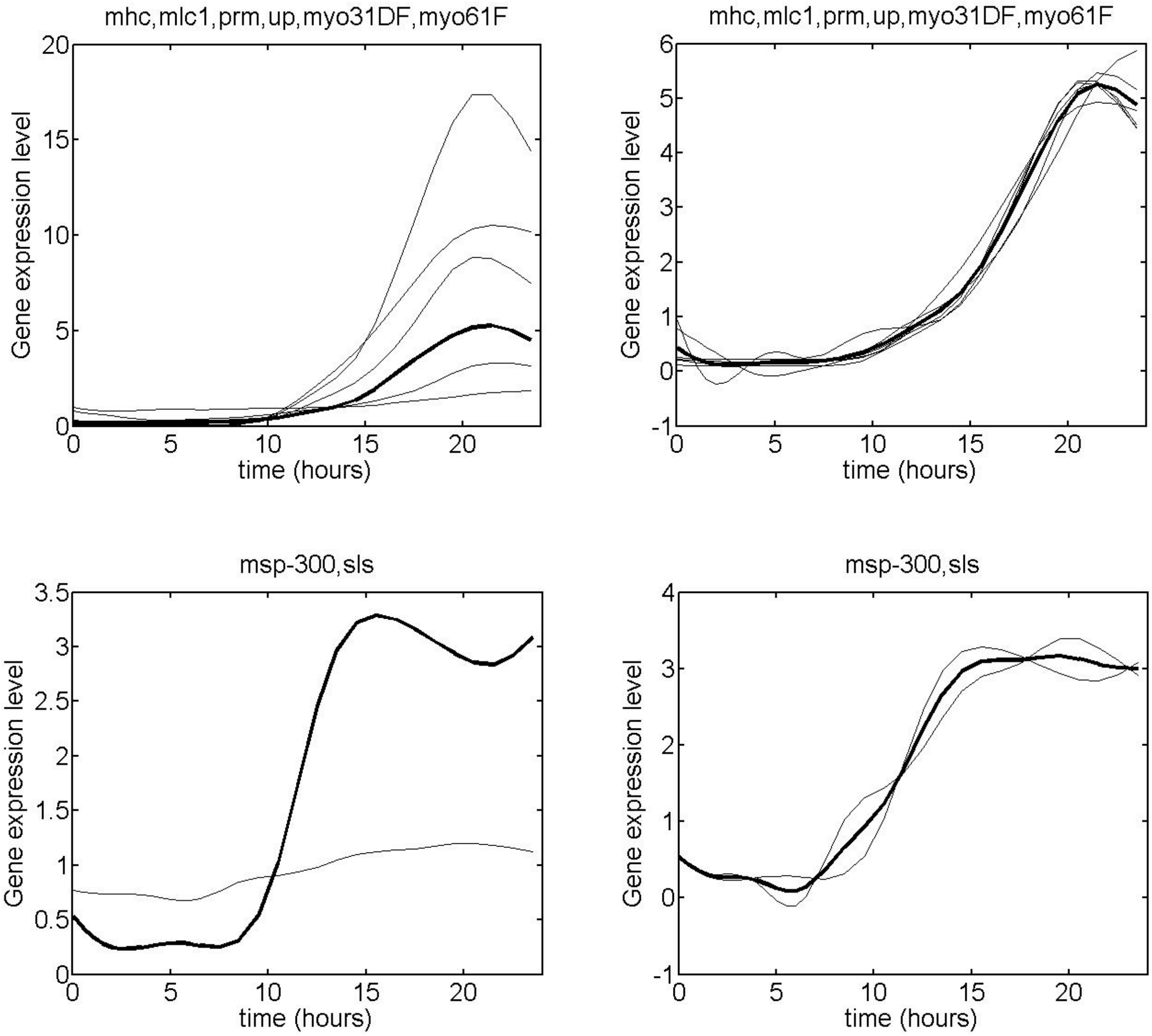

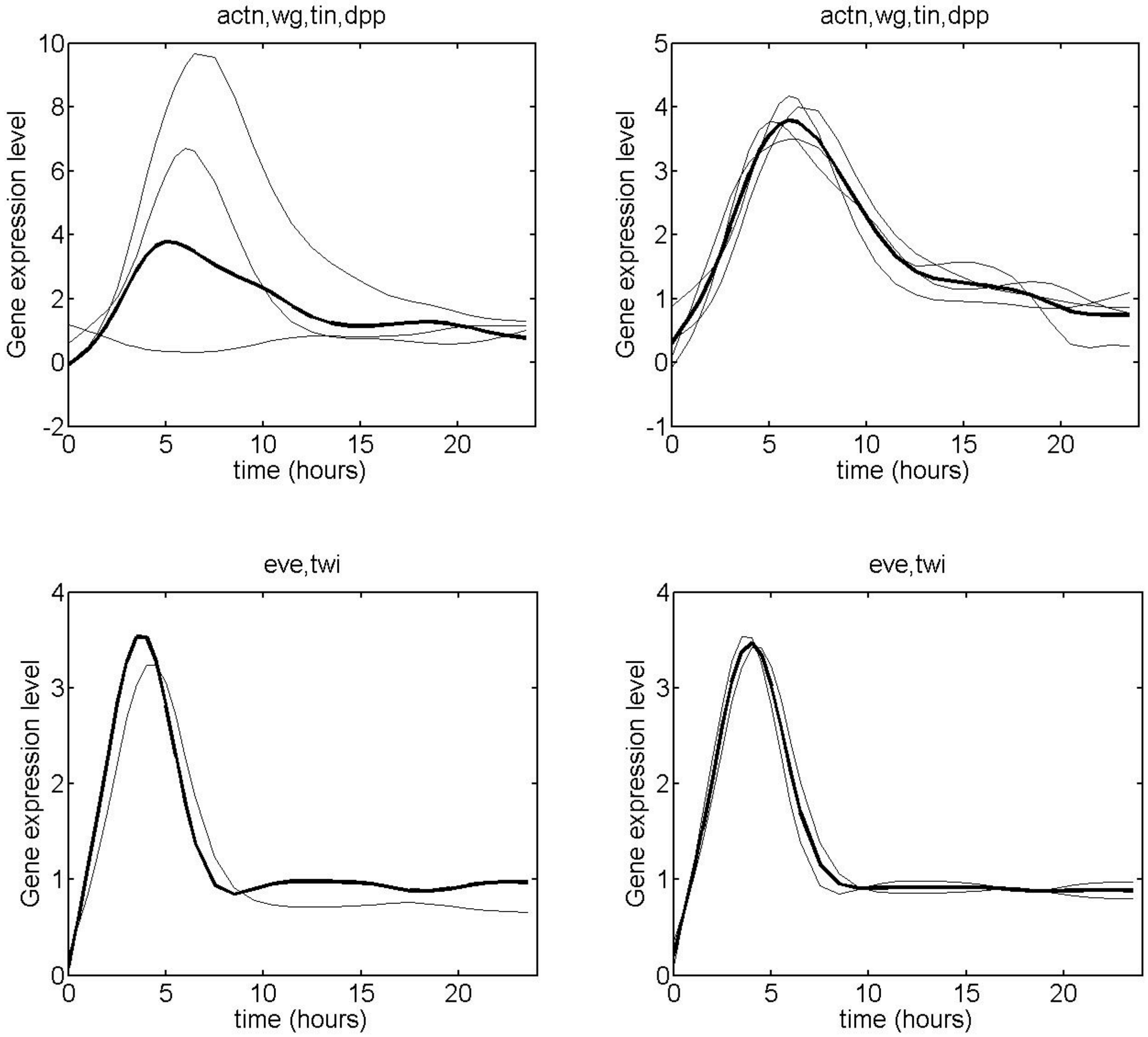
actn,wg,tin,dpp
Gene expression level
time (hours)
actn,wg,tin,dpp
Gene expression level
time (hours)
eve,twi
Gene expression level
time (hours)
eve,twi
Gene expression level
time (hours)

Figure S2. *The average profile of the ten clusters, after scaling and translation so that each profile has a standard deviation of one and no profile is negative.*

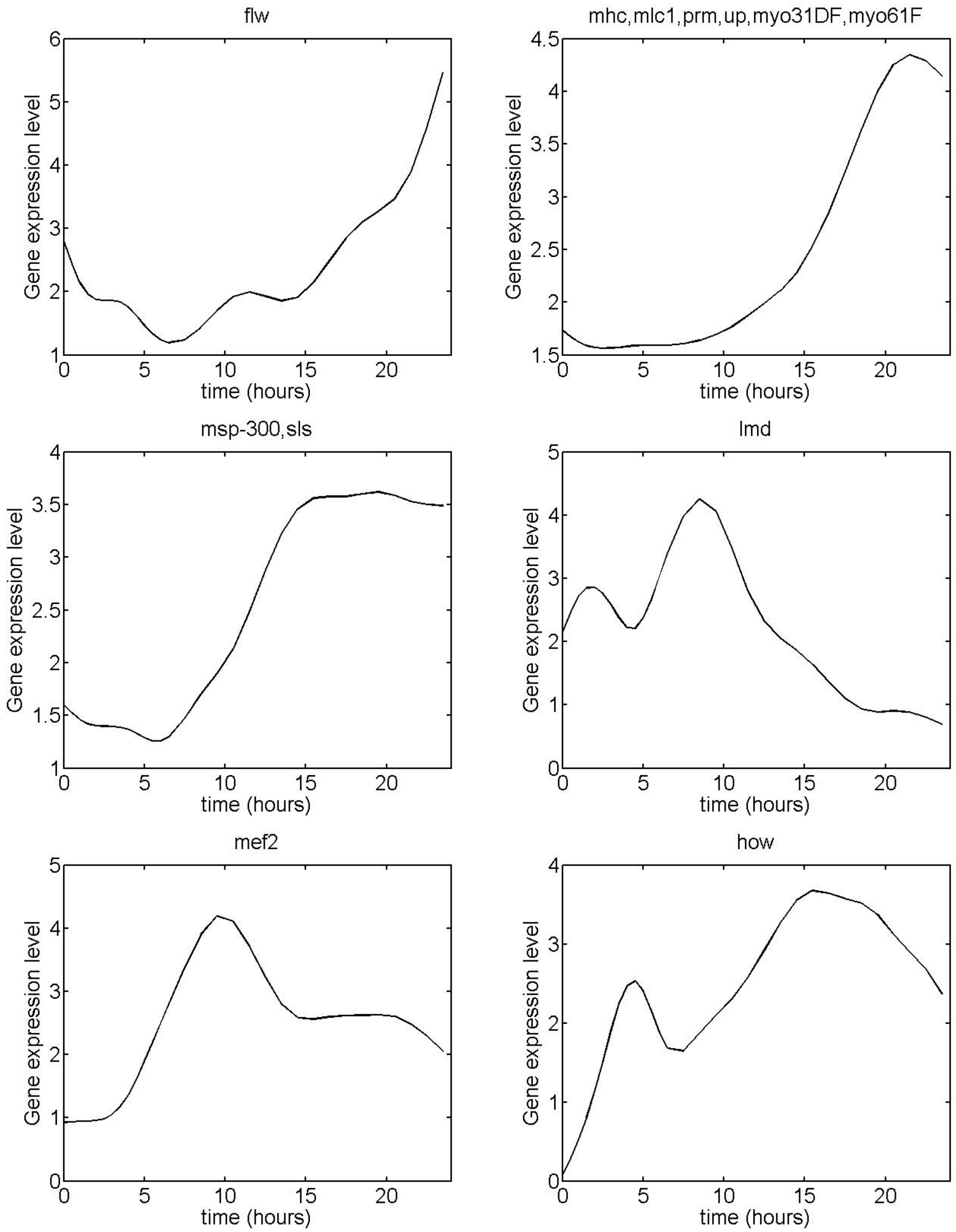

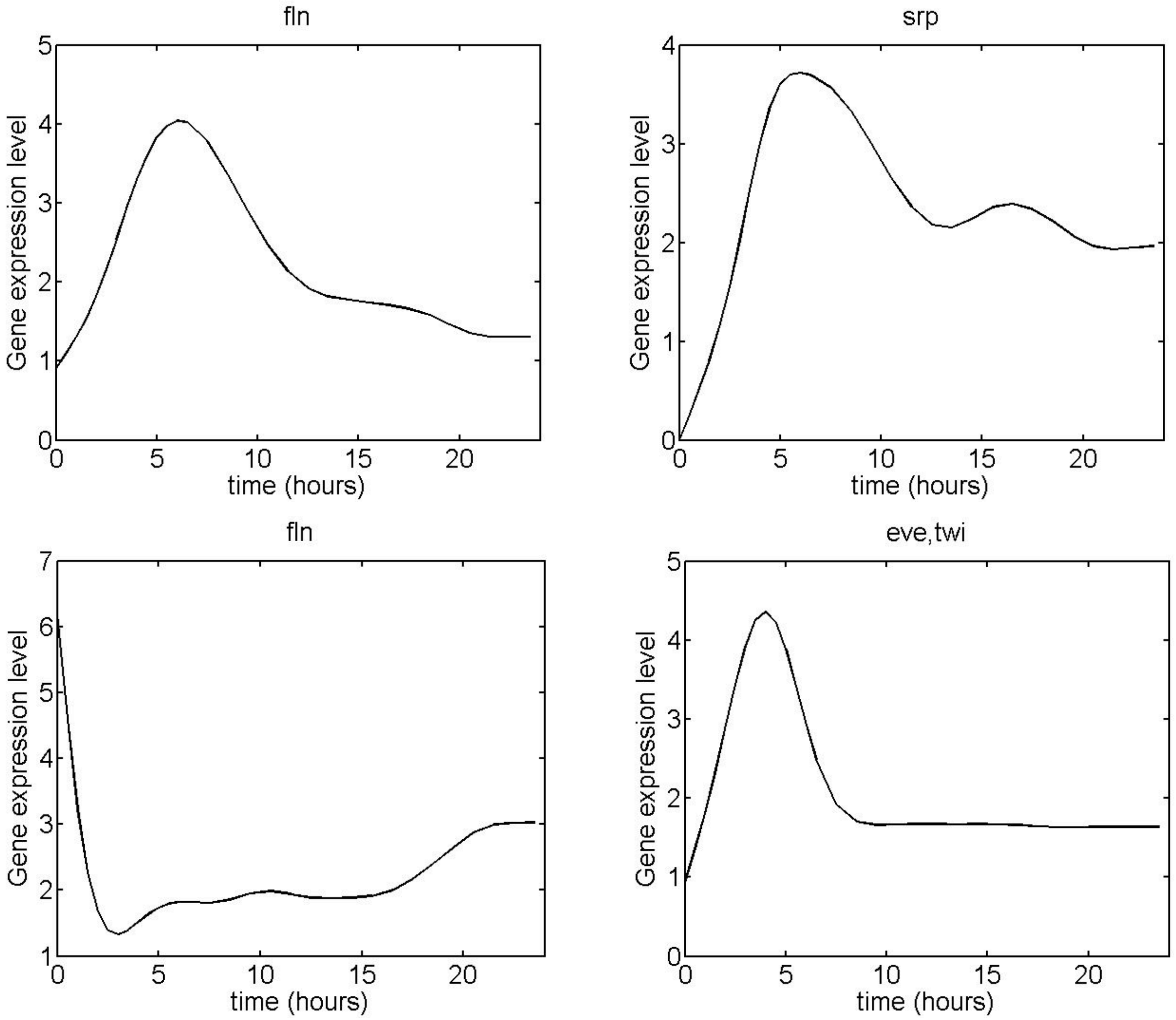
fln
srp
fln
eve,twi
Gene expression level
time (hours)

Figure S3: *Experimental and estimated gene expression profiles, with q=3. Dots: filtered and smoothed experimental data; dashed line:* $m_{NN}^{\exp}$ *; dotted line:* $m_{NC}^{\exp}$ *; solid line:* $m_{CN}^{\exp}$ *.*

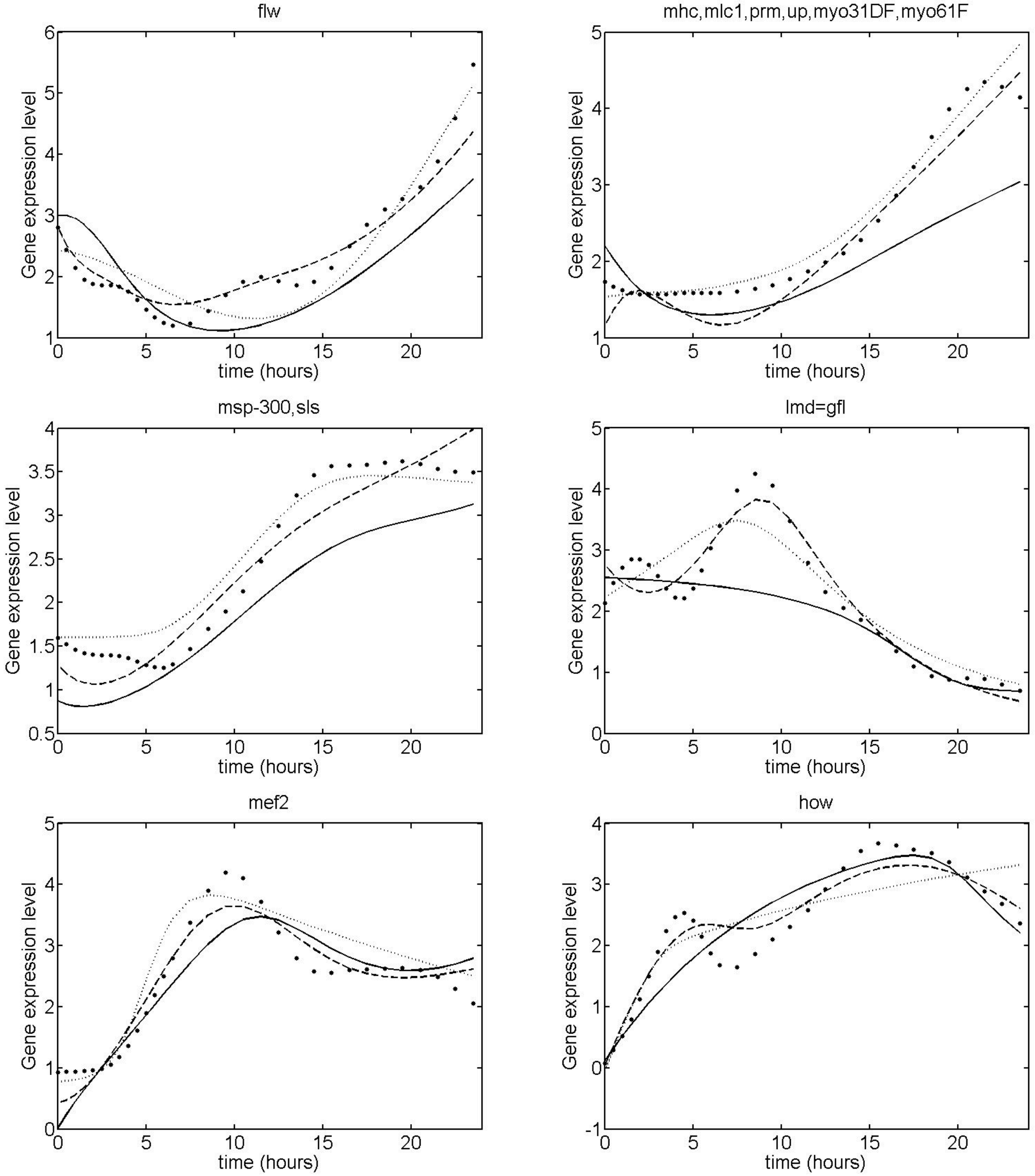

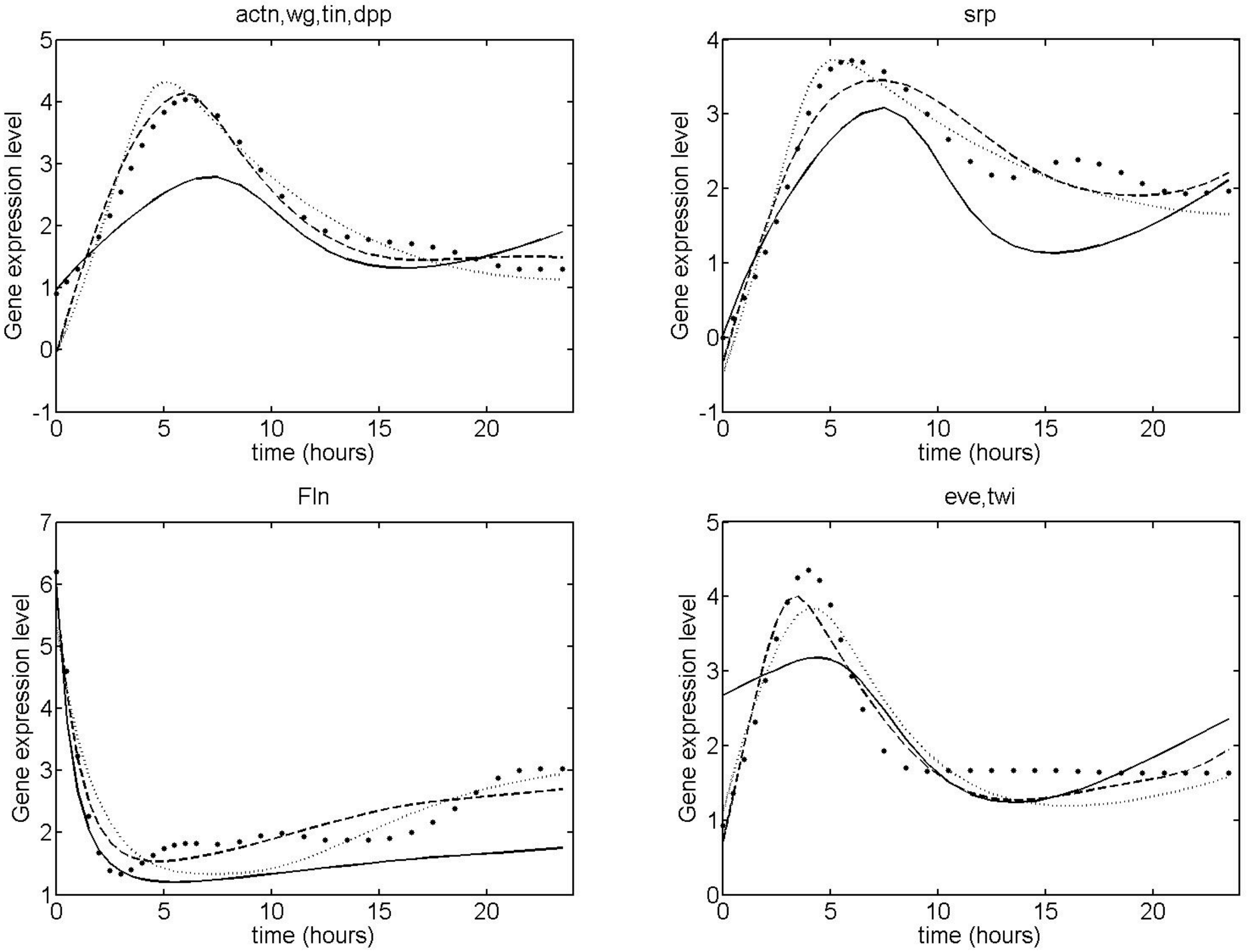

actn,wg,tin,dpp
srp
Fln
eve,twi
Gene expression level
time (hours)

Figure S4: *Experimental and estimated gene expression profiles for model* $m_{NN}^{\exp}$ *with q=2 before and after parameter reduction using the* Ψ_{ν} *procedure. Dots: filtered and smoothed experimental data; solid line: before parameter reduction; dashed line: after parameter reduction.*

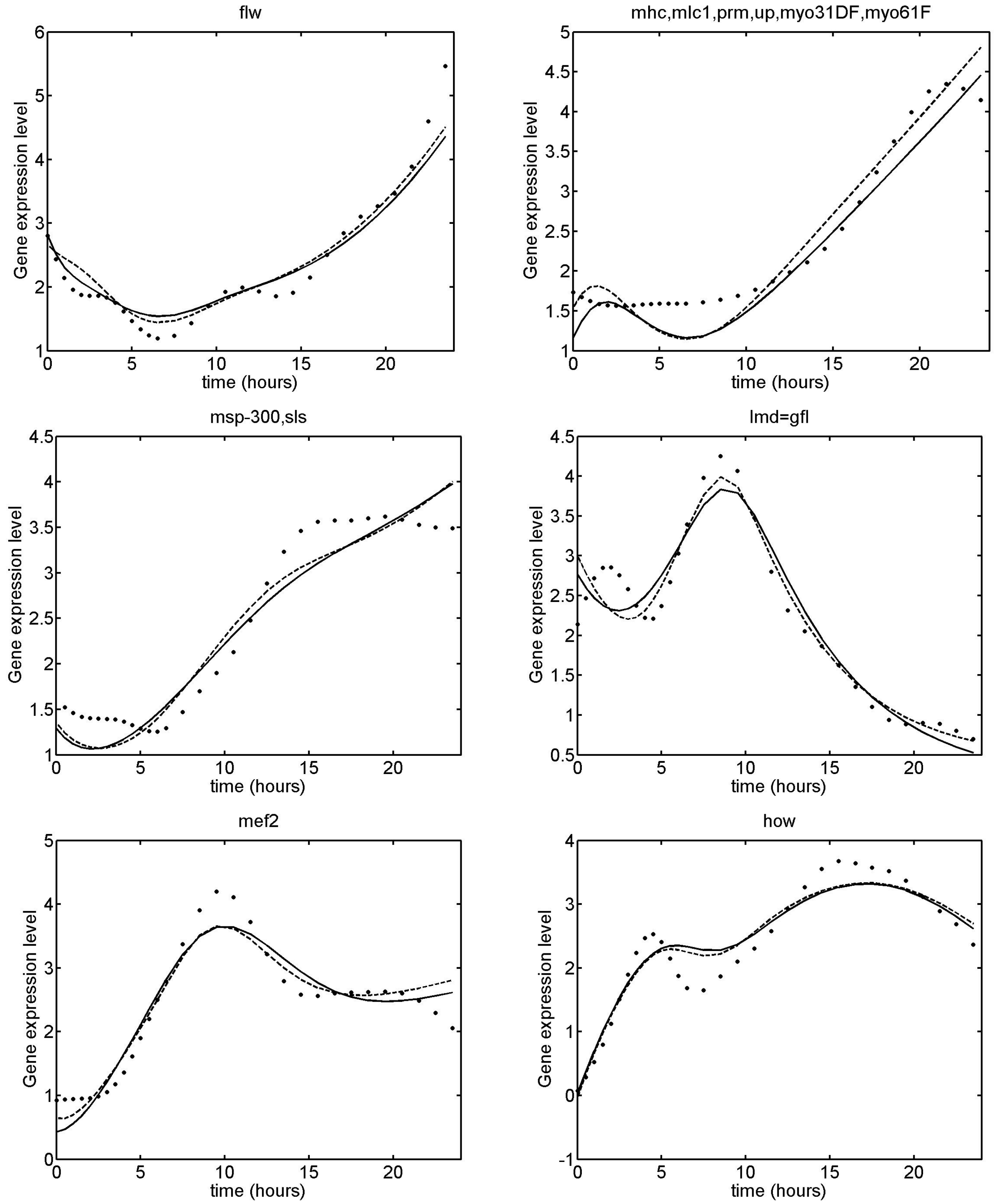

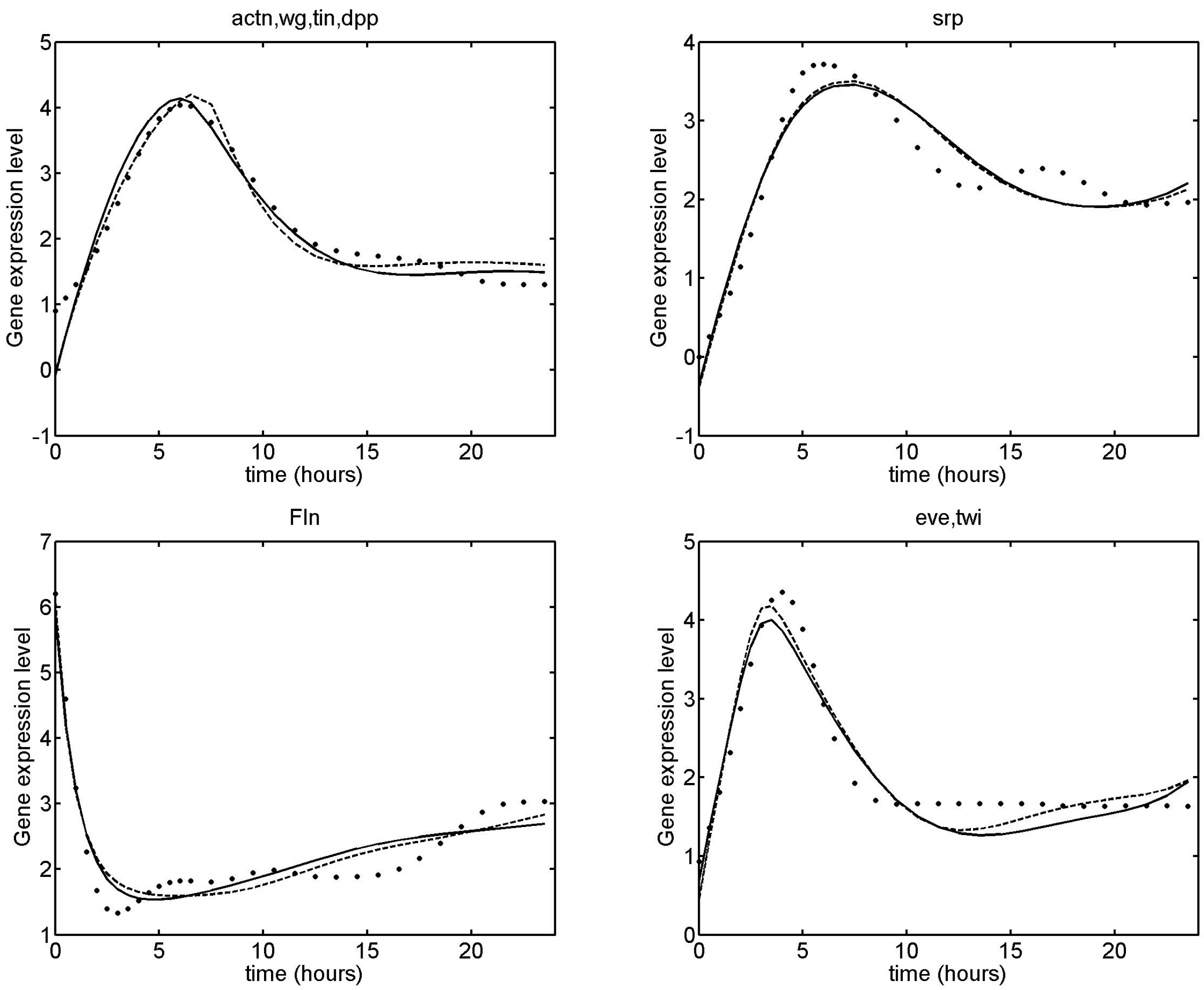

actn,wg,tin,dpp
srp
Fln
eve,twi
Gene expression level
time (hours)

Figure S5: *Experimental and estimated gene expression profiles for model* $m_{NN}^{\exp}$ *with q=3 before and after parameter reduction using the* Ψ_V *procedure. Dots: filtered and smoothed experimental data; solid line: before parameter reduction; dashed line: after parameter reduction*

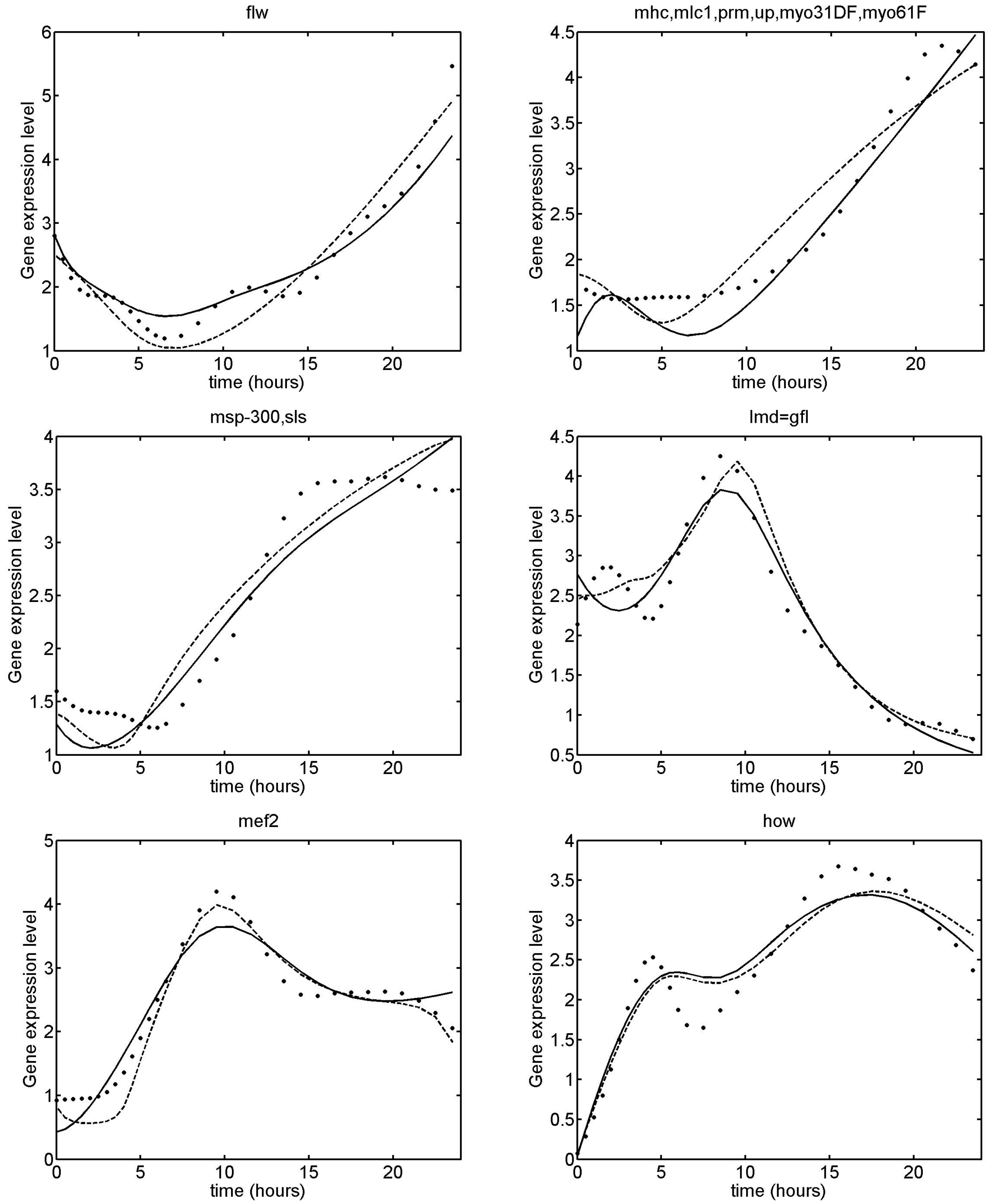

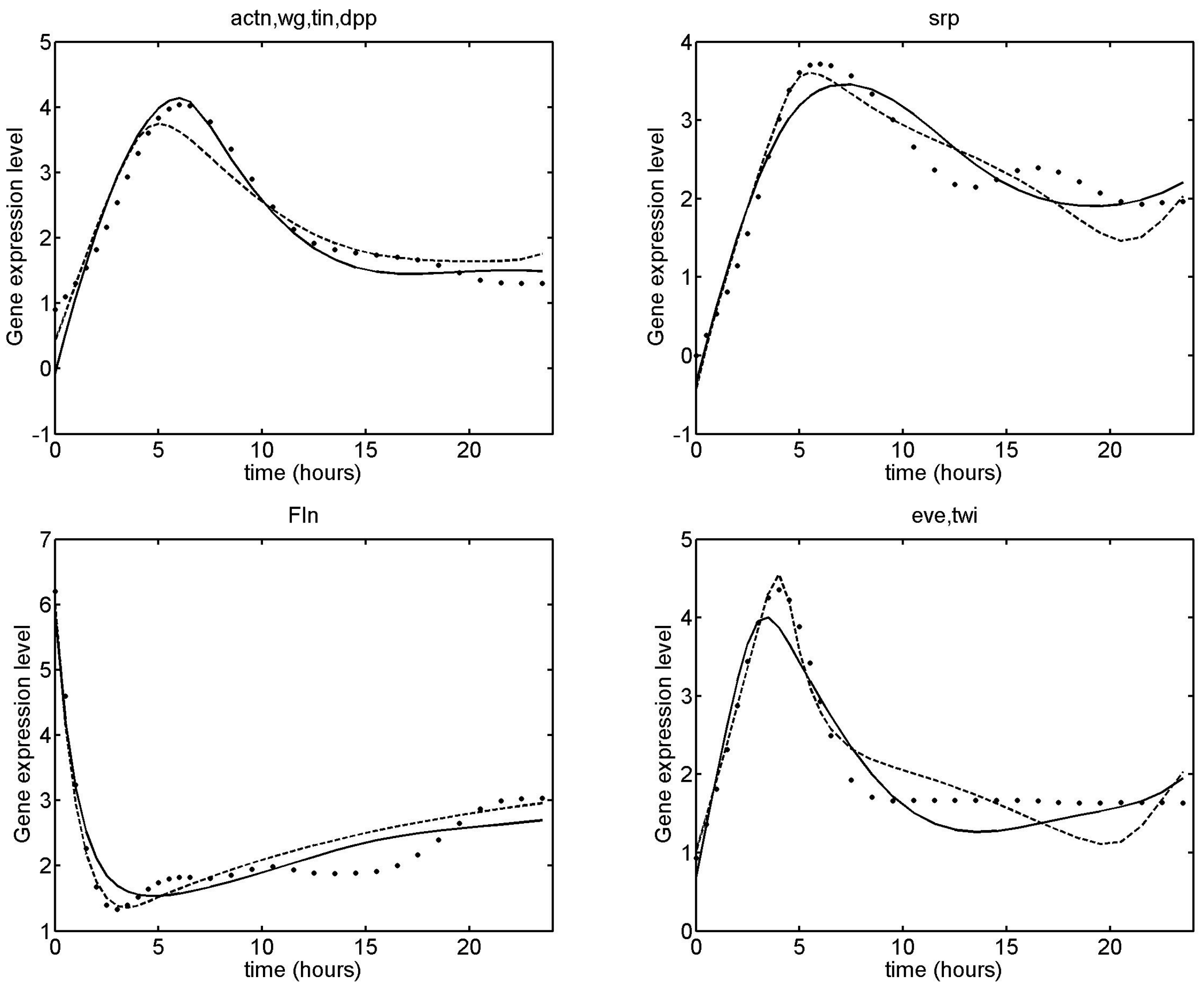

actn,wg,tin,dpp
srp
Fln
eve,twi
Gene expression level
time (hours)

Figure S6: *Experimental and estimated gene expression profiles for model* $m_{NN}^{\exp}$ *with q=4 before and after parameter reduction using the* Ψ_V *procedure. Dots: filtered and smoothed experimental data; solid line: before parameter reduction; dashed line: after parameter reduction*

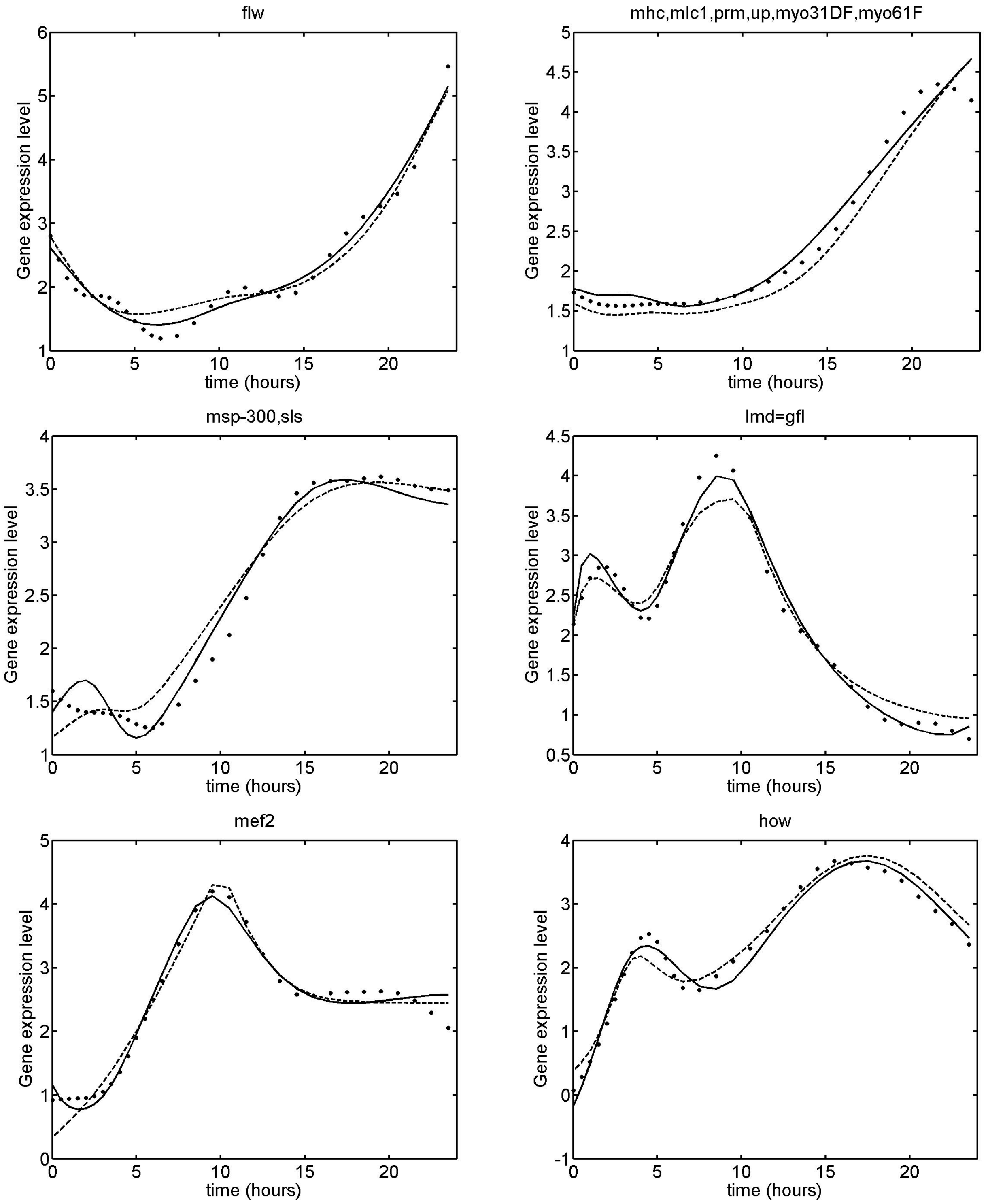

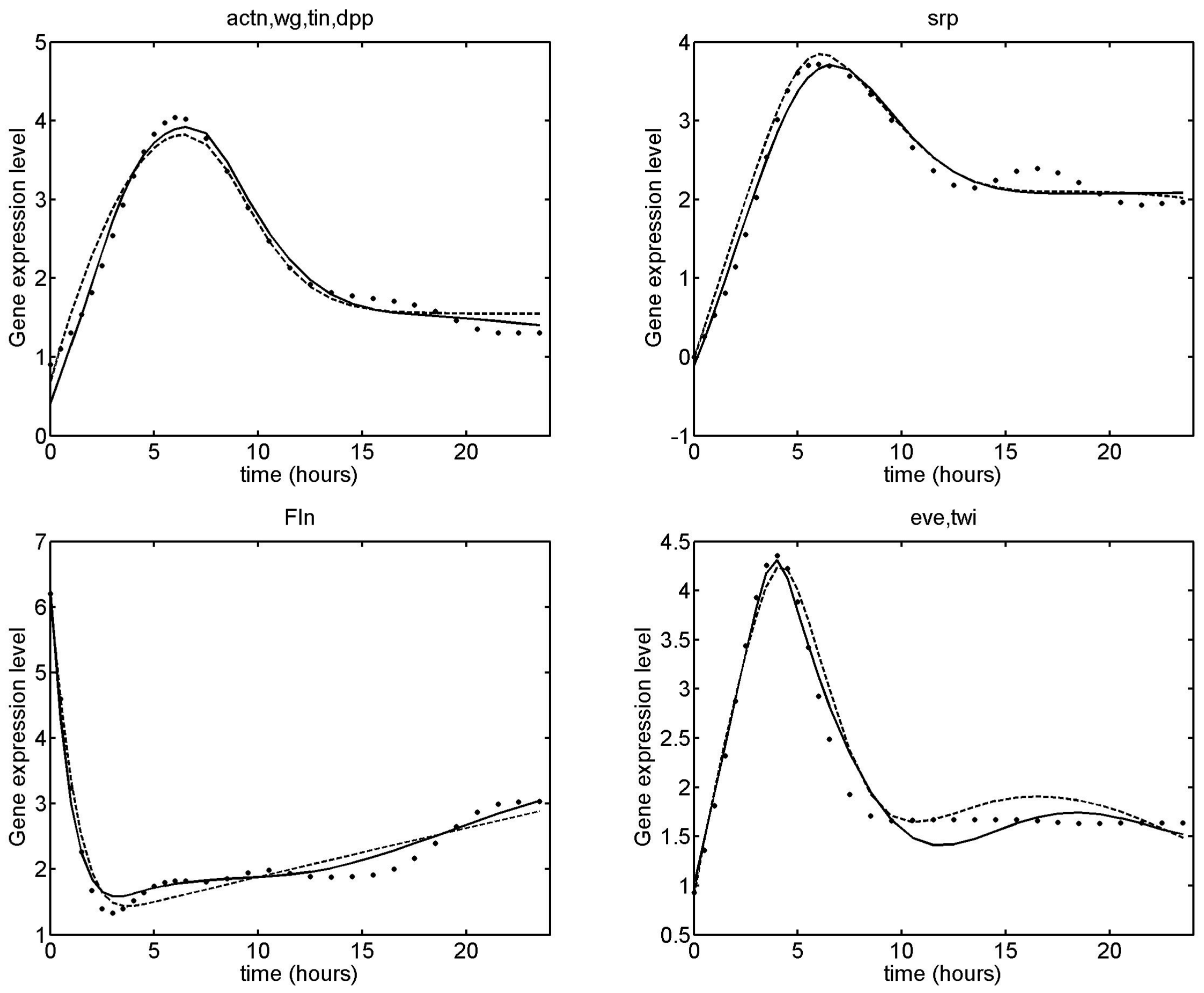

actn,wg,tin,dpp
srp
Fln
eve,twi
Gene expression level
time (hours)

Figure S7 : *Experimental and estimated gene expression profiles for model* $m_{NN}^{\exp}$ *with q=3 before and after parameter reduction using the* Ψ_v *procedure, when the 17 experimentally validated connections are imposed. Dots: filtered and smoothed experimental data; dashed line: before parameter reduction; solid line: after parameter reduction.*

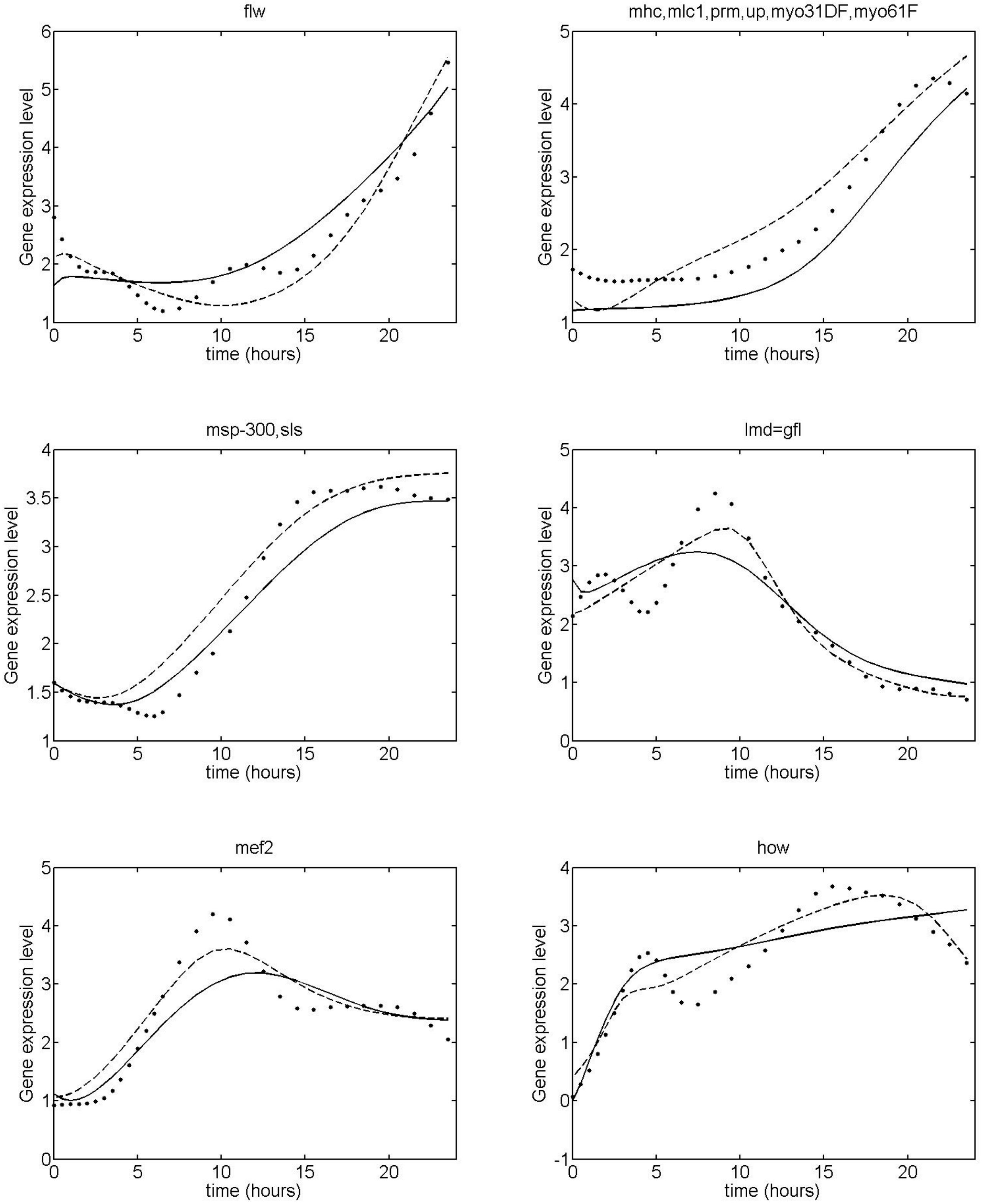

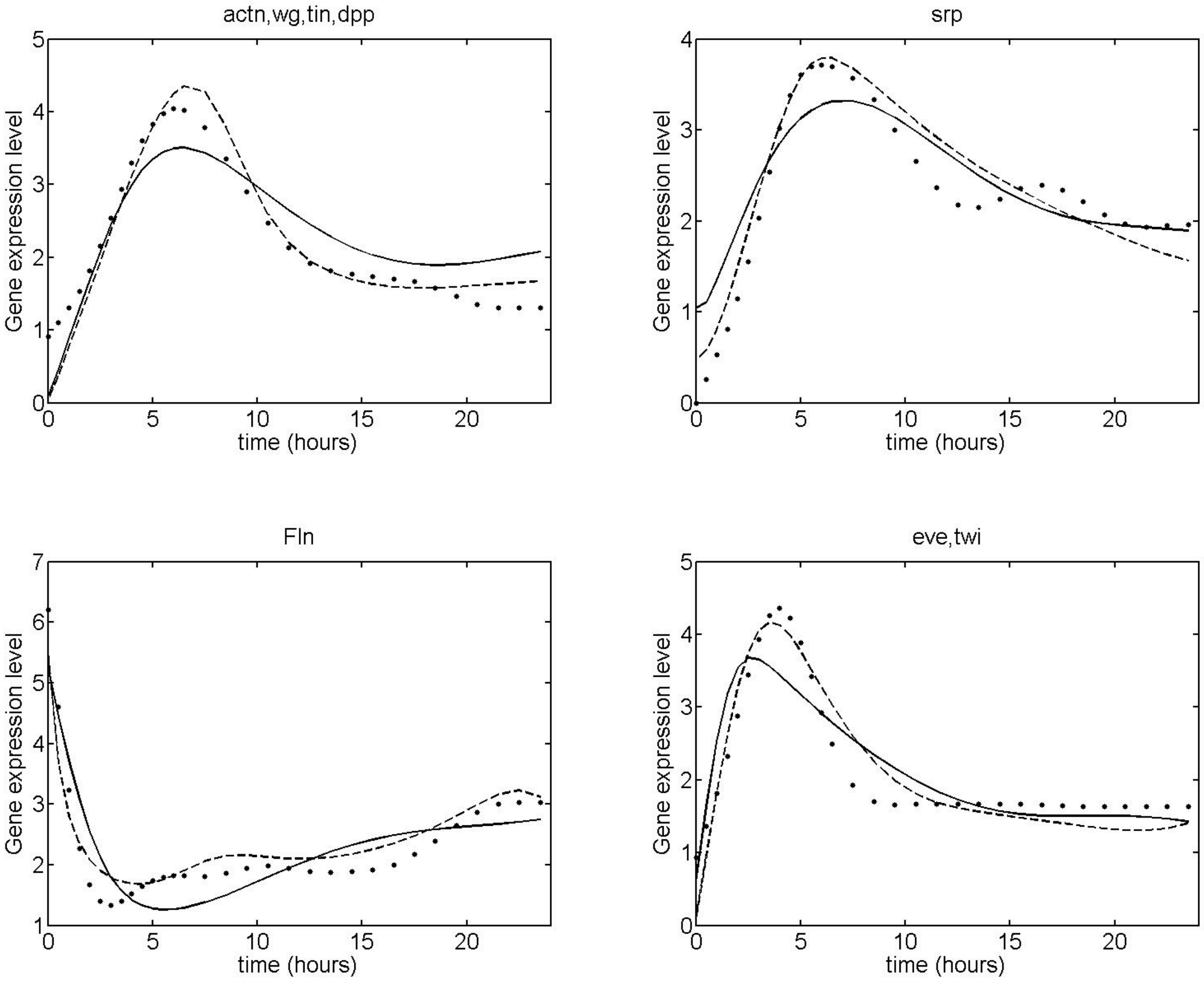

actn,wg,tin,dpp
srp
Fln
eve,twi
Gene expression level
time (hours)
